# Sciatica caused by spinal epidural abscess as the initial clinical presentation of colon cancer: a rare case report and review of literature

**DOI:** 10.1186/s12879-024-09159-6

**Published:** 2024-03-06

**Authors:** Kuan-Yu Lu, Wei-En Tung, Chang-Jung Chiang, Yueh-Ying Hsieh, Chia-Hsien Chen, Mei-Hui Lee, Min-Hsuan Yen, Po-Wen Lu, Lien-Chen Wu

**Affiliations:** 1 Department of Orthopedics, Shuang Ho Hospital, Taipei Medical University, New Taipei City, 23561 Taiwan; 2 Department of Orthopedic Surgery, School of Medicine, College of Medicine, Taipei Medical University, Taipei City, 110 Taiwan; 3 School of Biomedical Engineering, College of Biomedical Engineering, Taipei Medical University, Taipei City, Taiwan; 4 Division of Infectious Diseases, Department of Internal Medicine, Shuang-Ho Hospital, Taipei Medical University, New Taipei City, 23561 Taiwan; 5 Division of Colorectal Surgery, Department of Surgery, Shuang-Ho Hospital, Taipei Medical University, New Taipei City, 23561 Taiwan; 6 Division of Gastroenterology and Hepatology, Department of Internal Medicine, Shuang Ho Hospital, Taipei Medical University, New Taipei City, 23561 Taiwan; 7 Graduate Institute of Biomedical Materials and Tissue Engineering, College of Biomedical Engineering, Taipei Medical University, Taipei City, 110 Taiwan

**Keywords:** Colorectal cancer, *Streptococcus gallolyticus*, Spinal epidural abscess, Sciatica

## Abstract

**Background:**

Colorectal cancer is one of the most frequently diagnosed forms of cancer, and it is associated with several common symptoms and signs such as rectal bleeding, altered bowel habits, abdominal pain, anemia, and unintentional weight loss. Sciatica, a debilitating condition in which the patient experiences paresthesia and pain in the dermatome of associated lumbosacral nerve roots or sciatic nerve distribution, is not considered one of these. Here we present a case of colorectal cancer manifesting symptoms of sciatica alone.

**Case presentation:**

A 68-year-old male presented with progressive lower back pain radiating to his left thigh and calf over L5/S1 dermatome. Sciatica was suspected and initially underwent conservative treatment with analgesics. However, the symptoms progressed and MRI revealed an epidural abscess surprisingly. Surgical debridement was performed and pus culture isolated *Streptococcus gallolyticus.* Based on the strong association of *S. gallolyticus* with colorectal cancer, the presence of this pathogen prompted further tumor evaluation, even in the absence of the typical symptoms and signs. This investigation ultimately leads to the diagnosis of sigmoid adenocarcinoma.

**Conclusions:**

Although rare, sciatica caused by *S. gallolyticus* infection of the spinal epidural space may serve as the initial presentation of colorectal cancer. Physicians should be aware of the strong association between *S. gallolyticus* and colorectal cancer. Based on what we currently know about the condition; a thorough systematic assessment of occult neoplasia for patients with *S. gallolyticus* infection is recommended.

## Background

Colorectal cancer is the third most common malignancy and the second deadliest cancer worldwide [1]. Almost all cases of colorectal cancer are identified during diagnostic colonoscopy for suspicious signs and symptoms (80%); asymptomatic, routine screening (11%); or incidental finding at an acute abdomen emergent admission (7%). The common symptoms include rectal bleeding, altered bowel habits (narrowing, constipation, intermittent diarrhea, tenesmus), abdominal or back pain, anemia, and unintentional weight loss. Sciatica as the initial presentation of colorectal cancer is rare. Sciatica is a debilitating condition in which the patient experiences paresthesia and pain in the dermatome of associated lumbosacral nerve roots, usually caused by compression from intervertebral disc or other hypertrophic structures in the spinal column, rarely caused by epidural abscess. Spinal epidural abscess is an uncommon pyogenic infection located within the epidural space, often caused by *Staphylococcus aureus* and sometimes by *Streptococcus*. *Streptococcus gallolyticus*, formerly known as *Streptococcus bovis*, is an opportunistic pathogen sometimes cause bacteremia and infective endocarditis. Much evidence has accumulated that supports a specific association of *Streptococcus gallolyticus* with colorectal cancer. This article presents a rare case of a patient with symptoms of sciatica and was later diagnosed of colon cancer resulted from a tumor workup prompted by the isolation of *Streptococcus gallolyticus* from pus culture.

## Case report

**Fig. 1:**
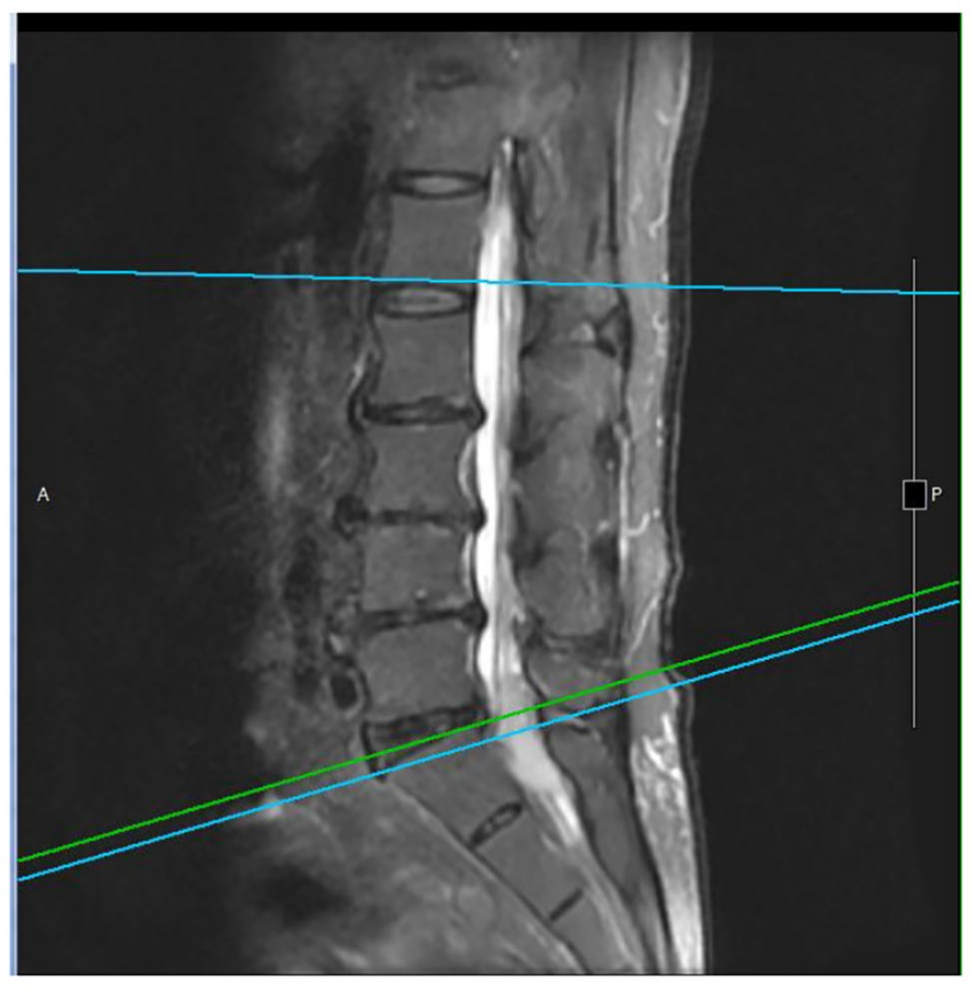
Indicates the L5-S1 level of the spinal epidural abscesses

**Fig. 2:**
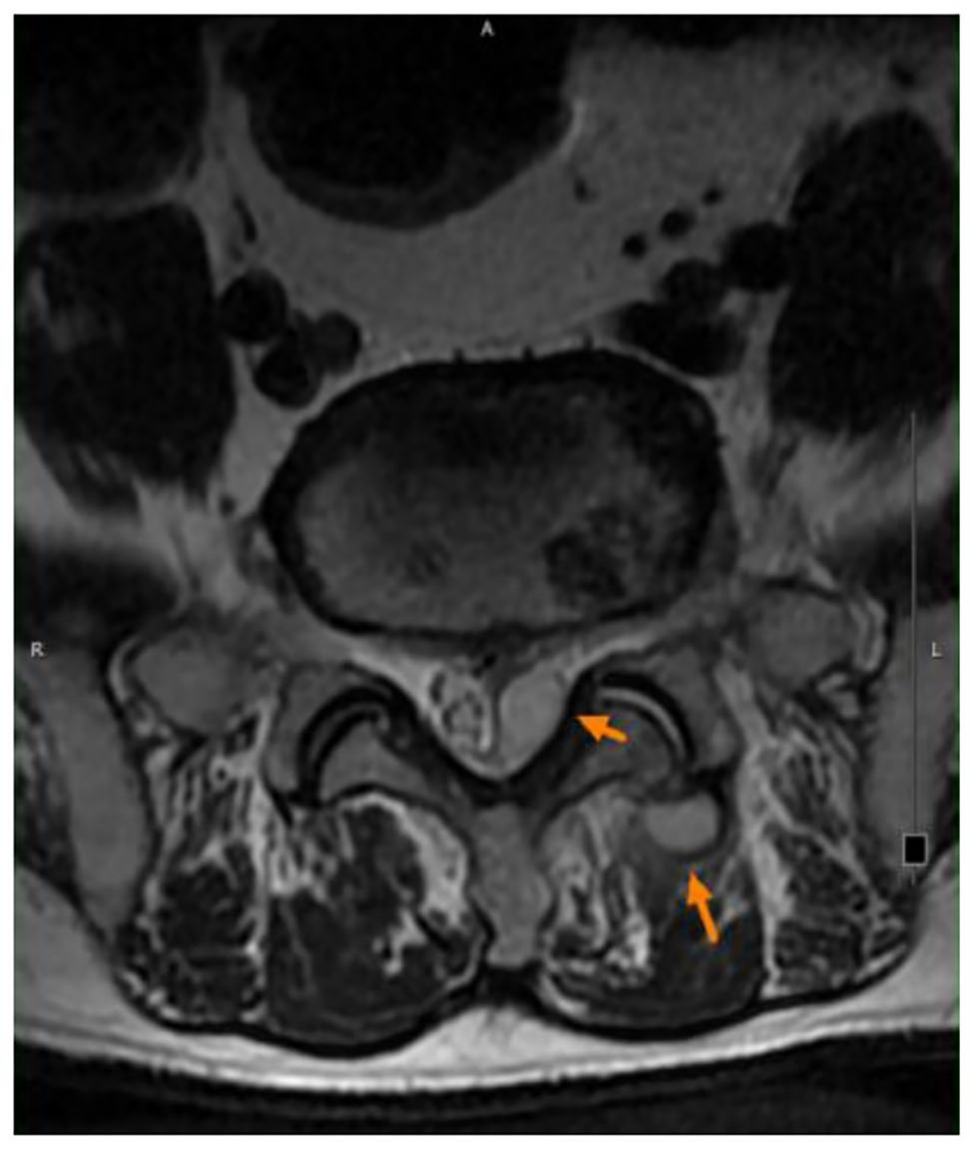
Shows the hyperintense signal of the masses in the T2-weighted image

**Fig. 3:**
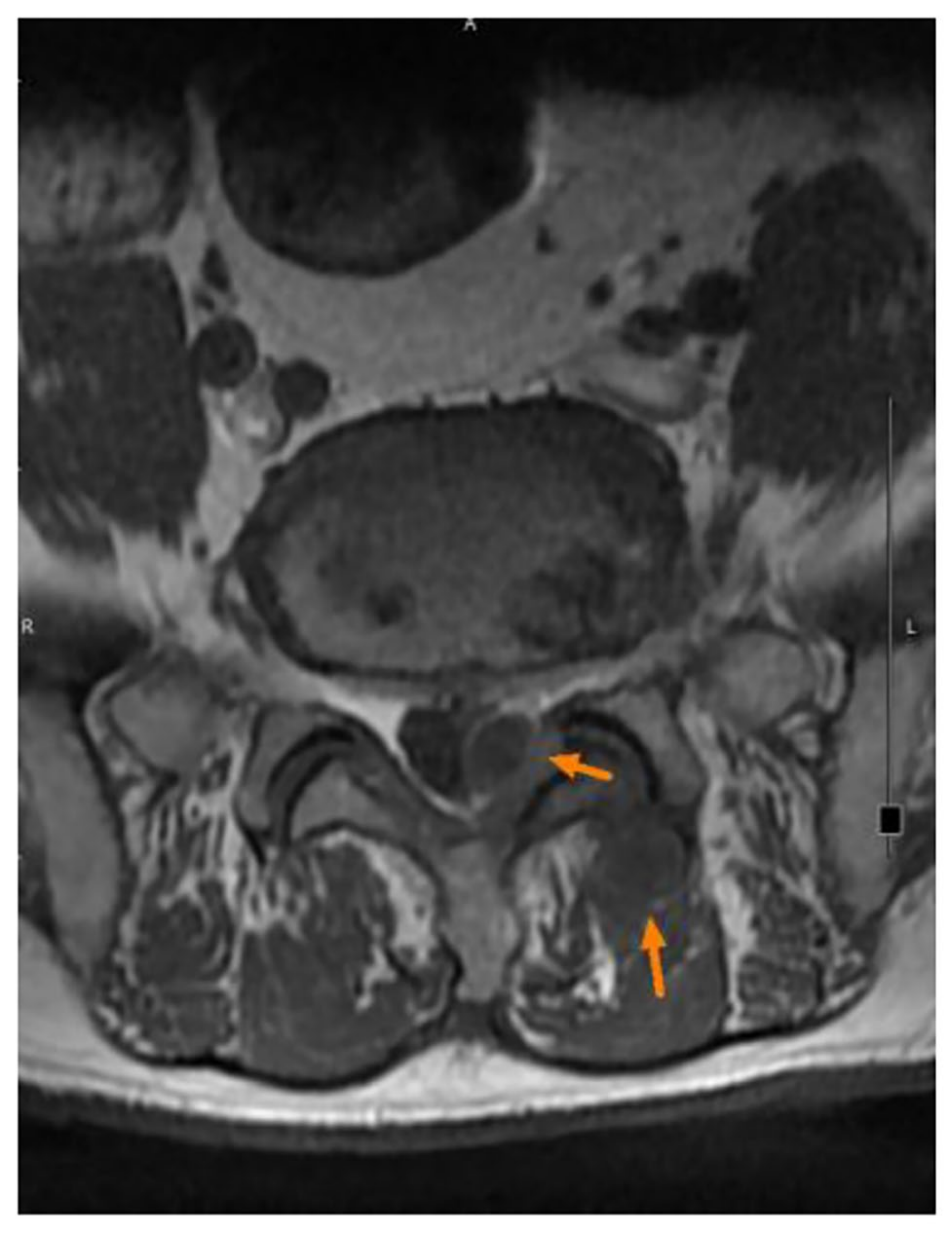
Shows the hypointense signal of the masses in the T1-weighted image

**Fig. 4:**
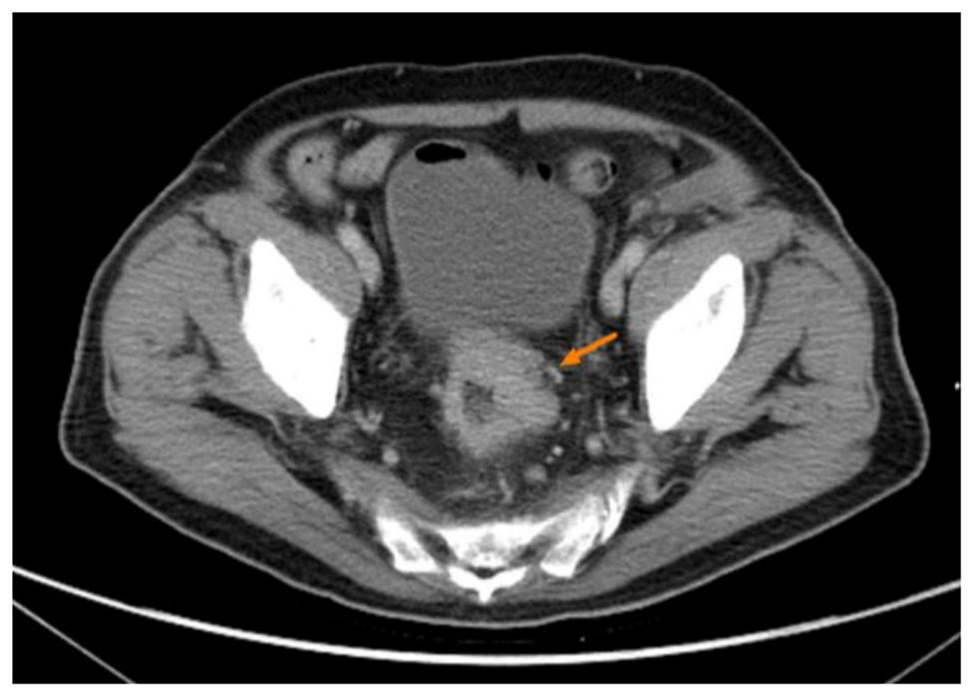
Displays the tumor found by using contrast CT

**Fig. 5:**
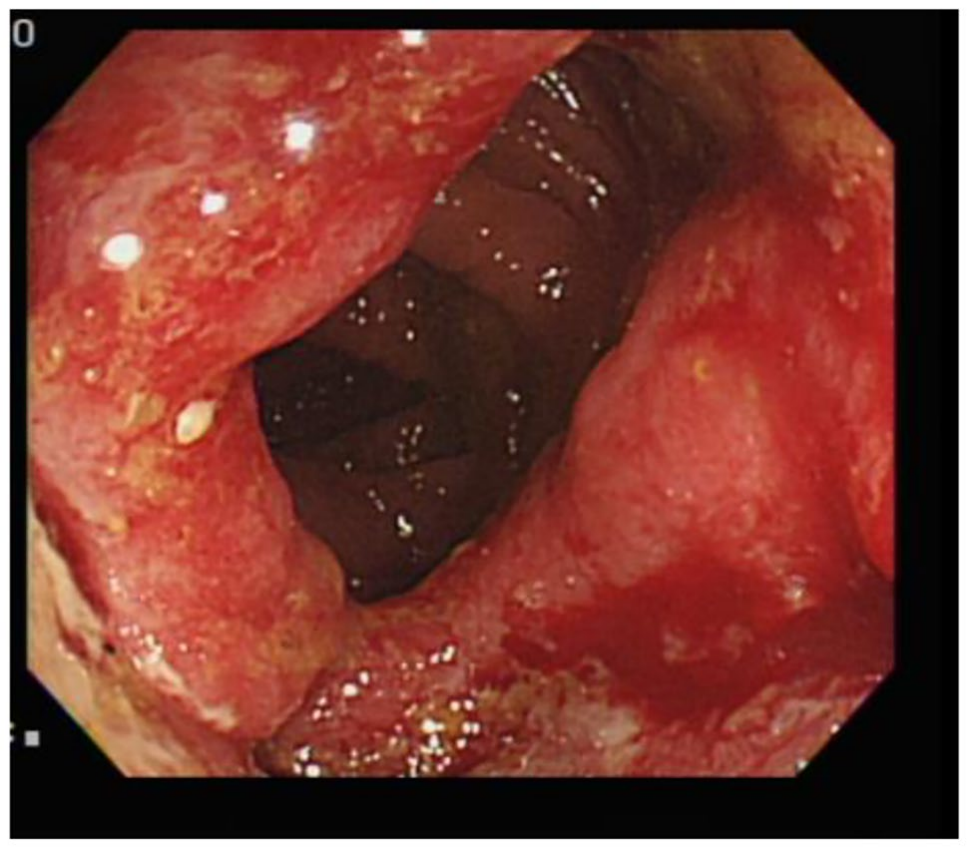
Displays the sigmoid tumor found during colonoscopy

A 68-year-old man with type 2 diabetes mellitus, hypertension and chronic obstructive pulmonary disease presented to our out-patient department with a progressive sharp lower back pain associated with left lower extremity numbness lasting more than 2 months. The symptoms were exacerbated by standing and walking and relieved by rest. Physical examination revealed midline lumbosacral tenderness with paresthesia radiating to his left thigh and calf over L5/S1 dermatome. L-spine plain films showed mild lumbar spondylosis with marginal spur formation and decreased disc height, which might imply degenerative disc disease that causing the symptoms. Nerve conduction velocity test and electromyography showed bilateral lumbosacral polyradiculopathy or sensorimotor polyneuropathy of lower limbs. Sciatica with diabetic polyneuropathy were suspected and conservative treatment with pain control, blood sugar control and rehabilitation were prescribed. However, after 3 months of treatment, the symptoms gradually progressed to involve his right leg and the patient complaint about claudication and bilateral leg weakness that he needed to walk with a walker. Physical exam in the clinic showed decreased bilateral leg muscle strength (Medical Research Council scale 4). Under the impression of sciatica refractory to conservative treatment, MRI without contrast was then arranged, which revealed spinal stenosis at L4/L5/S1 level and, surprisingly, an abnormal epidural mass at L5-S1 and a mass at the paraspinal area (Fig. 1, 2 and 3). These masses had well demarcated margins, and they displayed hypointense and hyperintense signals in the T1-weighted image and T2-weighted image, respectively. Abscesses or tumors were suspected on the basis of the images. He denied previous infection elsewhere, previous spinal surgery, injection, acupuncture and constitutional symptoms such as weight loss, headache, prolonged fevers, fatigue, dyspnea, and malaise. He was then admitted for further evaluation. The values for hemoglobin level, white blood cell count, CRP level, ESR and HbA1C from the initial laboratory study were 7.1 g/dL, 12.9 k/μL, 0.73 mg/dL, 67 mm/hr, and 9.4% respectively. Anemia was microcytic (MCV 73.1 fL) and the iron profile revealed iron deficiency anemia with superimposed chronic inflammation (serum iron 11 μg/dL, ferritin 85.54 ng/mL, TIBC 244 ug/dL, transferrin 223 mg/dL). The stool was soft and brown and he denied constipation and change of bowel habit. Digital rectal exam and stool occult blood test were unremarkable. Due to progression of his neurological symptoms, posterior decompression was performed on the 2nd day from admission. Epidural abscess at L5/S1 with adhesion of ligamentum flavum and dura, partial bony destruction of lamina and facet joint were noted in the operation. Posterior decompression with laminotomy of L5-S1, sequestrectomy of infected lamina and debridement were performed. The pus culture isolated *Streptococcus gallolyticus.* Ceftriaxone 2 g Q12H was administered and the sensitivity report later on showed susceptible. No microorganisms were isolated from the blood cultures, and no fever was noted before or after the operation. Transthoracic echocardiography revealed no valvular vegetation. Prompted by the well-established association between *S. gallolyticus* and colorectal cancer, a tumor workup was performed. Elevated tumor markers were noted: CEA level at 9.5 ng/mL (0.0–5.0) and CA199 level at 144.3 U/mL (0.0–37.0). The CT scan revealed a 5 cm tumor at the sigmoid colon extending to the sigmoid rectal junction (Fig. 4) with four enlarged lymph nodes around the inferior mesenteric artery. A colonoscopy located one circular ulcerative tumor at the sigmoid colon (Fig. 5). The biopsy revealed adenocarcinoma. The colorectal surgeon was consulted and a laparoscopic lower anterior resection was performed. The final pathology stage was determined to be pT3N2bMx, stage IIIC and the histologic grade was G2 (moderately differentiated). Notably, the patient had no family history of cancer and denied experiencing the common symptoms of colorectal cancer (weight loss, recent changes in bowel habits, and tarry or bloody stool). He tolerated the surgeries well and the neurologic symptoms gradually recovered. CBC/DC, CRP and ESR returned within normal ranges in a regular monitor. He was discharged after 19 days of hospitalization and was referred to hematology department for chemotherapy. He completed the treatment course and no focal recurrence nor distant metastasis was detected in a 3-year follow up period.

## Discussion and conclusions

*Streptococcus gallolyticus*, formerly known as *Streptococcus bovis*, belongs to the group D streptococci, a large group of phenotypically diverse bacteria known as the *S. bovis*/*S. equinus* complex. It had been appreciated since 1951 that the relationship between group D streptococci–induced endocarditis and colorectal cancer was first established by McCoy and Mason [2]. However, the detailed species associations were not well understood at that time. With the advancement of laboratory analysis, group D streptococci was further divided into seven divisions in 2015 based on multilocus sequence typing data: *Streptococcus gallolyticus* subspecies *gallolyticus* (*Sgg.*), *S. gallolyticus* subsp. *macedonicus, S. gallolyticus* subsp. *pasteurianus, Streptococcus infantarius* subsp. *infantarius, Streptococcus lutetiensis, Streptococcus alactolyticus* and *Streptococcus equinus* [3]. Much evidence has accumulated that supports a specific association of *Sgg.* with infective endocarditis and colorectal cancer [4]. 

*Streptococcus gallolyticus* subspecies *gallolyticus* (*Sgg.*) was first isolated from Koala feces, most probably for its ability to degrade tannins, which present in eucalyptus leaves [5] and owing its name due to its capacity to decarboxylate gallate, an organic acid derived from tannins hydrolysis [6]. It is a commensal bacteria of the gastrointestinal tract of different herbivores and is also identified in several animal species (e.g. bovine, pigeon, chicken, pig). *Sgg.* can also be found outside the animal host as a saprophyte and could be detected in the environment (e.g. dust and manure) [7]. It is detected in human gastrointestinal tract with varying prevalence from 2.5 to 62.5% of healthy humans [8]. A Rural residency and animal contact were risk factors further supporting the zoonotic potential transmission pathway. However, the accurate transmission route is not well understood. Direct, indirect transmission, zoonotic potential and oral fecal transfer were proposed [7]. *Sgg.* has genes involved in carbohydrates, tannases and bile salt degradation, conferring the bacterium the ability to survive in the gut [9]. *Sgg.* has pili that enable them to attach and colonize to the host tissues. Special pilus operon had been confirmed as virulence factor that mediate *Sgg.* binding to collagen types I and IV [10], the major structural component of human heart and the basal lamina layer underneath epithelial tissue, respectively. Of note, colonic tumors display higher levels of collagen IV compared to normal tissues [11]. Together, the results described a propensity tumor site colonization and how they initiate infective endocarditis. Over the last 6 decades, multiple epidemiological studies have confirmed the association of *Sgg.* infection and colorectal cancer. As shown in literature that *Sgg.* has been present in 47–85% of colorectal cancer cases [12, 13]. This association constitutes a strong recommendation for systematic assessment of occult neoplasia in patients with *S. gallolyticus* infections [4]. 

An interesting question regarding *Sgg.* is whether they are a consequence of altered host mucosal environment or if the bacteria themselves are oncogenic. Studies have demonstrated that *Sgg.* plays both roles [8, 14, 15] and two working models were proposed. Firstly, *Sgg.* acts as a passenger bacterium. In pre-neoplastic epithelium, activation of the Wnt pathway leads to the downregulation of bile acids transporter, resulting in accumulation of bile acids. This in turn activates a specific “bacteriocin” enabling *Sgg.* to kill other commensals. Thus benefit *Sgg.* for its own multiplication. This dysbiosis can contribute to the development of colorectal cancer. Secondly, *Sgg.* acts as a driver bacterium. High colonization of *Sgg.* in pre-malignant epithelium can induce specific inflammatory responses (e.g. IL-1, IL-8 and COX-2) [16] and increased cell proliferation, which is associated with upregulation of β-catenin levels and its oncogenic downstream targets (c-Myc and cyclin D) [17], thus accelerating transformation from pre-malignant to malignant cell. Recent studies give us some more insights to the pathogenicity. Taddese et al. in 2021 shows that *Sgg.* have the potential to upregulate the *CYP1A* and *ALDH1* genes expression in colonic epithelial cells that encode phase I biotransformation enzymes (e.g. cytochrome P450) responsible for the detoxification or bio-activation of toxic compounds, miserably toxify some toxic substrates (e.g. polycyclic aromatic hydrocarbons in food and environmental pollutants) into chemical intermediates that are more genotoxic than their precursors, resulting in more susceptibility of the intestinal cell to DNA damaging events [18]. Taylor et al. in 2021 identified a locus in *Sgg.* that encodes a putative type VII secretion system contributing to gut colonization and the development of colon tumors [19]. Teh et al. in 2023 further characterized one of the type VII secretion system effector proteins, named herein TelE, exhibiting their toxic activity of promoting the loss of membrane integrity [20]. Pasquereau-Kotula et al. demonstrate the oncogenic role of *Sgg.* in a murine model. Full proteome and phosphoproteome analysis revealed that 164 proteins and 725 phosphorylation sites were differentially regulated by *Sgg.*, associating with activation of multiple cancer-related signaling pathways such as MAPK, mTOR and integrin/ILK/actin, thus accelerating tumor development [21]. Taylor et al. identified a chromosomal locus in *Sgg.* designated as “the *Sgg.* pathogenicity-associated region” as a critical pathogenicity determinant. Deletion of this locus significantly reduced *Sgg.* adherence to colorectal cancer cells and abrogated the ability of *Sgg.* to stimulate cancer cell proliferation [22]. Taken together, whether *Sgg.* is a cause or consequence of colorectal cancer is not clearly understood and the relationship is quite complex.

Concerning the prognosis of patient with *Streptococcus gallolyticus* infection and colorectal cancer, a study showed that early-stage adenomas have more incidence with the presence of *S. gallolyticus* than later-stage carcinomas [23], which is in line with another report showing that 68% of colorectal cancer patients with positive *S. gallolyticus* infection have Stage 1 or 2 colorectal cancer compared to only 32% for stages 3 or 4 [24]. Another multi-center comparative study showed that, compared with patients with *Clostridium septicum* bacteremia, those with *S. gallolyticus* bacteremia had less invasive carcinomas, smaller tumor size, lower overall colorectal neoplasm related mortality rate. Of note, most of them presented as occult colorectal neoplasm [25]. The association between the presence of *S. gallolyticus* and their early stages of colorectal cancer might aid in detecting disease sooner, thus preventing further deterioration of diseases [26]. 

Spinal epidural abscess is an uncommon pyogenic infection located within the epidural space. It is a serious condition with high morbidity (such as quadriparesis and paraparesis) and mortality if left untreated. Four phases of the clinical presentation have been described as fever and back pain, then radicular pain and nuchal rigidity, then neurological deficits, and finally paralysis [27]. The most common age for spinal epidural abscess is 50 to 70 years, and men are more frequently affected than women [28]. Spinal epidural abscess can occur from secondary to direct bacterial inoculation of the spinal column or through hematogenous spread of bacterial pathogens. Direct inoculation can occur in patient with recent spinal surgery, spinal injections, and skin defects close to the spinal column. Hematogenous spread occurs when a bacterial infection presents in another part of the body and subsequently spreads through the vascular system, resulting in gainning access to the spinal column [28]. Intravenous drug use, recent trauma, alcohol use, and immunocompromise (such as diabetes and HIV) are risk factors for spinal epidural abscess. The most common bacterial pathogen is *S. aureus*; however, *Streptococcus* and gram-negative bacteria are also possible sources of spinal epidural abscess [29]. A systematic review of 12 studies comprising 1099 patients revealed diabetes (27%) as the most commonly associated medical comorbidity; the lumbar spine (48%) as the most common location of involvement; back pain (67%) followed by motor weakness (52%) as the most common presenting symptoms and 60% of patients was managed surgically [29]. Although the typical hallmark triad comprised of back pain, fever, and neurological deterioration is observed only in 8 to 15% of cases [30]. 

The uniqueness of this case was that the patient presented with only symptoms of sciatica caused by epidural abscess. He did neither present with systemic inflammatory symptoms, nor exhibited signs of colorectal cancer, with the possible exception of anemia. The patient denied having any recent trauma, spinal surgery, injections, and IV drug use. No wound was noted on his back during physical examination. Therefore, we inferred that the spinal epidural abscess was formed through hematogenous spread of *S. gallolyticus* and that the source was the gut colonization of the bacteria over the tumor mucosa. However, whether *S. gallolyticus* was a cause or a consequence of the colorectal cancer remains a mystery. After he diagnosed with colorectal cancer, we reviewed his clinical record and medical history. Body temperature of 37.4’C under regular use of acetaminophen was noted. The use of analgesic might mask his fever and delay the diagnosis of epidural abscess. Moreover, despite reported defecation 2–3 times every day with normal texture and color, there was a decrease in overall stool output. Owing to poor controlled DM (HbA1C 9.4%), our patient might be in a relatively immunocompromised condition which might make the *S. gallolyticus* hematogenous spread more probable. Physicians should be aware of infection, even in the absence of fever, especially in patient with regular use of analgesics. Furthermore, although rare, keep in mind of epidural abscess as a differential diagnosis of back pain. The limitation of this report is that the subspecies analysis of *S. gallolyticus* was not available in our institute and we didn’t perform test to check whether *S. gallolyticus* did colonize at the cancer mucosa.

Although rare, sciatica caused by *S. gallolyticus* infection of the spinal epidural space may serve as the initial presentation of colorectal cancer. Physicians should be aware of the strong association between *S. gallolyticus* and colorectal cancer. Based on what we currently know about the condition; a thorough systematic assessment of occult neoplasia for patients with *S. gallolyticus* infection is recommended.

## Data Availability

Not applicable.

## Abbreviations

*Sgg*: *Streptococcus gallolyticus* subspecies *gallolyticus*
